# Editorial: Diversifying the STEM fields: From individual to structural approaches

**DOI:** 10.3389/fpsyg.2022.1113227

**Published:** 2023-01-05

**Authors:** Rodolfo Mendoza-Denton, Colette Patt, Adrienne R. Carter-Sowell

**Affiliations:** ^1^Psychology Department, University of California, Berkeley, Berkeley, CA, United States; ^2^Division of Math and Physical Sciences, University of California, Berkeley, Berkeley, CA, United States; ^3^Psychology Department, University of Oklahoma, Norman, OK, United States

**Keywords:** STEM, broadening participation, diversity, graduate education, professoriate, National Science Foundation (NSF), AGEP

The articles in this collection represent one snapshot of the work conducted under the auspices of the National Science Foundation (NSF). Many, though not all, contributions were presented at the NSF AGEP (Alliances for Graduate Education and the Professoriate) 2017 conference, which was held at the Clark Kerr campus of the University of California, Berkeley. The contributions represent a range of approaches–from theoretical to empirical to programmatic- to addressing equity and representation in graduate education. We firmly believe that each of these approaches richly contributes to the national conversation around broadening participation in STEM, as no one approach is going to give us a full picture of viable solutions and processes. Theoretical work may not translate well to applied settings, and real-world contingencies help elucidate and sharpen theoretical advances. Programmatic work impacts and benefits scholars in real-time, and often serves as a lifeline to underrepresented students navigating the road to the a doctoral degree. At the same time, programmatic work is conducted in settings with multiple factors simultaneously affecting outcomes, thus necessitating empirical work to help tease out and elucidate the processes that affect student success. Empirical work, however, is itself limited by its reductive and controlled nature; it requires both theoretical and programmatic work to remain relevant in the field. Together—theoretical, empirical, and programmatic approaches—help advance the field more than any single approach possibly could.

In this volume, we have also sought to represent a range of lenses through which to approach broadening participation in STEM. Broadening participation, almost by definition, means being open to different ideas and different ways of knowing, and of being critical and reflective about the very way we go about achieving our goals. Broadening participation means that not everybody who participates in a common endeavor will have the same worldviews, or the same understandings of what scholarship, mentorship, and even science might mean. Our efforts too easily become hegemonic if we do not remain attuned to the assumptions and invisible norms that govern our practices. It is as important for us to remain open to critiques of how we do things, as it is for us to remain vigilant of the critiques we offer for other approaches. Broadening participation cannot be seen as a one-way entryway through which people walk through, fully conforming to the norms, traditions, and standards of the fields they are being invited to participate in. Rather, we must recognize that people are meant to change the field itself, to shape it, and to bring new questions and perspectives along with them. In doing so, our science grows more complex, more complete, and more collaborative.

## History

Since 1998, the National Science Foundation (NSF) has invested more than $380 M in alliance-based approaches to increasing the diversity of the faculty in the sciences, technology, engineering, and mathematics fields (STEM).

Begun as the Minority Graduate Education (MGE) program, this initiative has supported universities in changing their institutional, departmental, and organizational cultures. The NSF, at the start, provided funding to higher educational institutions focused on designing and implementing practices that could result in significant increases in recruitment, retention, degree conferral and career (especially academic) entry in the number of African American, Hispanic, and Native American students receiving doctoral degrees in the sciences, mathematics and engineering. Eight universities were awarded nearly $2.5-million MGE grants each and the American Association for the Advancement of Science (AAAS) was charged with evaluating the effectiveness of this new program. This first group of MGE institutions to receive awards was: University of Puerto Rico; Howard University; University of Missouri-Columbia; University of Alabama-Birmingham; Georgia Institute of Technology; University of Michigan; Rice University; and University of Florida.

In 2002 the program was renamed the Alliances for Graduate Education and the Professoriate (AGEP). An additional 18 AGEP Alliances were awarded prior to 2008. The AAAS analysis, in 2010, documented a 21% increase in the average annual number of historically underrepresented minority (URM) PhD recipients in STEM at 19 of the 26 AGEP awardee institutions included in the sample. Further solicitations for NSF AGEP project proposals followed (National Science Foundation, 2012, 2014, 2016). While the long-term goal remained the same, namely, to increase the number of historically underrepresented minority STEM faculty, each call for proposals indicated a shift in expectations and requirements. For example, after the first two cycles, the NSF moved away from direct funding of designated graduate student fellowships for URMs and toward creating alliance-based strategies or “models” for change that might lend themselves to adoption at other institutions in higher education. From the 2012–2016 calls for proposals, more than 112 institutions of higher education partnered in one or more NSF AGEP alliance. Characteristics noted for funded institutions include the Basic Carnegie Classification of Institutions of Higher Education (Indiana University Center for Postsecondary Research, 2021) as well as designations for minority serving institutions (U.S. Department of Education, 2020). All institutions are located within the continental United States. Two-thirds of the partnering institutions have doctoral programs with high or very high research activity according to the Carnegie Basic Classification. The other third comprises schools focused on degrees at the associate's, baccalaureate, and master's program levels, tribal colleges, and a few professional doctoral programs. The number of partners in each alliance ranged from two or three to more than nine. Five institutions, The State University of New York at Stony Brook, Texas A&M University, Tuskegee University, the University of California, Berkeley, and the University of Maryland Baltimore County, lead consecutive or multiple NSF AGEP alliance projects. From 2012–2018, the NSF supported 27 alliances. Since 1998, NSF has funded more than 350 awards to 130 different institutions/organizations. AGEP has reached all 50 states and the Commonwealth of Puerto Rico, the District of Columbia and the Virgin Islands of the United States.

In 2012 a requirement to include social science and education research was added specifically to build the knowledge base about underlying issues, policies and practices that have an impact on the participation, transition, and advancement of URMs in the STEM fields.

By 2019, the funded AGEP projects, collectively, had generated a panoply of programmatic strategies and models, a range of approaches to evaluating their effectiveness, and a growing set of studies related to these efforts. The time was right to share the results among those working on AGEP projects, and beyond it to the community of social scientists interested in addressing the long-standing problems of underrepresentation of racial and ethnic historically minoritized groups in the professoriate. The University of California, Berkeley, hosted the first AGEP conference focused on sharing of social science research results in 2017, establishing a tradition with subsequent conferences held annually—including remotely during the pandemic years. Emerging from the normative context of the work to increase the number and representation of URM STEM faculty of the AGEP alliances, the social science research contributes rigorous documentation of the progress made by these projects, and data-informed suggestions about paths forward toward the long-term goals established by the NSF two and a half decades ago.

In 2021 the NSF issued a new AGEP solicitation, which continues the program's focus on increasing a racially and ethnically diverse STEM academic workforce. The new solicitation supports grants that address institutional changes in the systemic and organizational policies, practices, culture and climate that support equity and inclusion, and mitigate inequities, in the academic profession and workplaces. AGEP does this through two funding tracks: AGEP Catalyst Alliances and AGEP Institutional Transformation Alliances. All tracks require collaborative university and college teams to use an intersectional lens to promote systemic change that considers the intersection of race, ethnicity, gender and other social identities. The AGEP Catalyst Alliances track supports the design and implementation of one or more organizational self-assessment(s) to collect and analyze data that will identify inequities affecting the AGEP populations; pilot equity strategies as appropriate; and develop a five-year equity strategic plan for the AGEP populations. The AGEP Institutional Transformation track is designed to support the development, implementation, and evaluation of innovative systemic and institutional change strategies that promote equity for AGEP populations, within similar institutions of higher education. ITAs create permanent policy and practice changes that advance AGEP populations, and the project work is expected to be sustained after NSF funding expires.

## Author contributions

All authors listed have made a substantial, direct, and intellectual contribution to the work and approved it for publication.
